# Exposure to SARS-CoV-2 and Infantile Diseases

**DOI:** 10.1055/s-0043-1768699

**Published:** 2023-05-02

**Authors:** Darja Kanduc

**Affiliations:** 1 Department of Biosciences, Biotechnologies and Biopharmaceutics, University of Bari, Bari, Italy

**Keywords:** SARS-CoV-2 spike gp, molecular mimicry, peptide sharing, cross-reactivity, immunologic imprinting, autoimmune, infantile diseases

## Abstract

**Background and Aim** Immune response against severe acute respiratory syndrome coronavirus 2 (SARS-CoV-2) in newborns and children after prophylactic immunization is currently a relevant research topic. The present study analyzes the issue by examining the possibility that the anti-SARS-CoV-2 immune responses are not uniquely directed against the virus but can—via molecular mimicry and the consequent cross-reactivity—also hit human proteins involved in infantile diseases.

**Methods** Human proteins that—if altered—associate with infantile disorders were searched for minimal immune pentapeptide determinants shared with SARS-CoV-2 spike glycoprotein (gp). Then, the shared pentapeptides were analyzed for immunologic potential and immunologic imprinting phenomena.

**Results** Comparative sequence analysis shows that: (1) numerous pentapeptides (namely, 54) are common to SARS-CoV-2 spike gp and human proteins that, when altered, are linked to infantile diseases; (2) all the shared peptides have an immunologic potential since they are present in experimentally validated SARS-CoV-2 spike gp-derived epitopes; and (3) many of the shared peptides are also hosted in infectious pathogens to which children can have already been exposed, thus making immunologic imprint phenomena feasible.

**Conclusion** Molecular mimicry and the consequent cross-reactivity can represent the mechanism that connects exposure to SARS-CoV-2 and various pediatric diseases, with a fundamental role of the immunologic memory and the history of the child's infections in determining and specifying the immune response and the pathologic autoimmune sequela.

## Introduction

Recently, researchers and clinicians called attention on the issue of vaccinating newborns and children against severe acute respiratory syndrome coronavirus 2 (SARS-CoV-2) to protect from coronavirus disease 2019 (COVID-19). Pros and cons have been examined and discussed in light of the following data:

- Children account only for 1.7 to 2% of the diagnosed cases of COVID-19. 1
- COVID-19 in children shows a milder disease course and better prognosis than adults. Mortality is extremely low. 2
- COVID-19 is deadlier for aged people than for other age groups. 3
- Severe manifestations of COVID-19 in adults comprehend dyspnea, respiratory failure, pneumonitis, thromboembolic events, cardiogenic shock, renal injury, ischemic strokes, encephalitis, and cutaneous eruptions. 4
- In contrast, severe manifestations of COVID-19 in children appear to be associated only with an uncommon, somewhat serious but tractable inflammatory disorder, that is, the so-called multisystem inflammatory syndrome in children (MIS-C). MIS-C is rarest and rarely is fatal. Indeed, Ergenc et al 5 reported that among 1,340 patients aged between 0 and 216 months and diagnosed with COVID-19, only 6 patients had MIS-C, which corresponds to a MIS-C incidence of 0.4%. None of the patients died. In parallel, Payne et al 6 reported that MIS-C incidence per 1,000,000 SARS-CoV-2 infections was 316.
- Moreover, and crucially, an analysis of the potential risk of autoimmune cross-reactivity 7 8 9 10 11 12 13 14 15 16 17 lacks while it should be compulsorily included in the benefit–risk assessment of SARS-CoV-2 vaccination in children.

Then, the present study aims at exploring (or excluding) the possibility that molecular mimicry and the consequent potential cross-reactivity may exist between SARS-CoV-2 and human proteins that are linked, when altered, to infantile diseases, so that targeting the viral antigen via vaccination might equate to hit human proteins linked to childhood pathologies.

## Materials and Methods

Peptide sharing between SARS-CoV-2 spike glycoprotein (gp) (NCBI, GenBank Protein Accession Id = QHD43416.1) from SARS-CoV-2 and human proteins related to childhood diseases was analyzed as previously detailed 9 10 using the pentapeptide as minimal immune determinant unit. Pentapeptides were used as sequence probes since a peptide grouping composed of five amino acid (aa) residues defines a minimal immune unit that can (1) induce highly specific antibodies and (2) determine antigen–antibody-specific interaction. 18 19 A library of 372 human proteins linked—when altered—to pediatric diseases was obtained from UniProt database ( www.uniprot.org ) 20 using the keyword “infantile.” The 372 proteins are listed in Supplementary Table S1 (online only).

CoV controls were as follows, with NCBI:txid number in parentheses: Middle East respiratory syndrome (MERS)-CoV (1335626); human (H) CoV-229E (11137); HCoV-NL63 (277944).

Methodologically, the SARS-CoV-2 spike gp primary aa sequence was dissected into pentapeptides offset each other by one residue (i.e., MFVFL, FVFLV, VFLVL, FLVLL, and so forth) and the resulting viral pentapeptides were analyzed for occurrences within the human proteins related to infantile diseases. Peptide Match and Peptide Search programs available at www.uniprot.org ^20^ were used.

The immunologic potential of the peptides shared between SARS-CoV-2 spike gp and proteins related to childhood diseases was analyzed by searching the Immune Epitope Database (IEDB, www.iedb.org/) ^21^ for immunoreactive SARS-CoV-2 spike gp-derived epitopes hosting the shared pentapeptides.

Finally, pentapeptides common to SARS-CoV-2 spike gp–derived epitopes and human proteins related to infantile diseases were additionally controlled for occurrences in the following bacterial pathogens listed with NCBI:txid number in parentheses: *Bordetella pertussis* (257313), *Corynebacterium diphtheriae* (257309), *Clostridium tetani* (212717), *Haemophilus influenzae* (71421), and *Neisseria meningitides* (122586).

## Results and Discussion

### Molecular Mimicry between SARS-CoV-2 Spike gp and Human Proteins Related to Infantile Diseases

Table 1 shows that SARS-CoV-2 spike gp shares a high number of minimal immune determinants (namely, 54) with 43 human proteins that associate with infantile disorders when altered, mutated, or, however, improperly functioning. The following points emerge from Table 1 :

**Table 1:** Peptide sharing between SARS-CoV-2 spike gp and human proteins related to infantile diseases

Peptides a	Human proteins, pathologies, and references b
TECSN	*ANTR2. Anthrax toxin receptor 2* Juvenile hyaline fibromatosis and infantile systemic hyalinosis 22
**GAGAAA**	*ARX. Homeobox protein ARX* Lissencephaly associated with abnormal genitalia 23
DIAAR	*AT1A2. Sodium/potassium-transporting ATPase subunit α-2* Alternating hemiplegia of childhood. Epilepsy 24 25
SFELL	*CLN6. Ceroid-lipofuscinosis neuronal protein 6* Seizures, dementia, visual loss, and/or cerebral atrophy 26
NSVAY	*CO1A1. Collagen α-1(I) chain* Osteogenesis imperfecta/Ehlers–Danlos' syndrome 27
TLLAL	*COX15. Cytochrome c oxidase assembly protein homolog* Microcephaly, encephalopathy, hypertrophic cardiomyopathy, lactic acidosis, respiratory distress, hypotonia and seizures 28
FLLKY	*CTNS. Cystinosin* Late-onset juvenile or adolescent nephropathic cystinosis 29
NLLLQ, VPVAI, AGTIT	*DPOG1. DNA polymerase subunit gamma-1* Juvenile-onset Alpers' syndrome and status epilepticus 30
SEPVL	*FRMD7. FERM domain-containing protein 7* Infantile nystagmus syndrome 31
EDLLF, **LQELGK**	*GLSK. Glutaminase kidney isoform, mitochondrial* Neonatal epileptic encephalopathy 32
SSVLN	*HCN1. Potassium/sodium hyperpolarization-activated cyclic nucleotide-gated channel 1* Infantile epileptic encephalopathy 33
YLQPR	*MTU1. Mitochondrial tRNA-specific 2-thiouridylase 1* Mitochondrial infantile liver disease 34
SLLIV	*NALCN. Sodium leak channel nonselective protein* Hypotonia, speech impairment, intellectual disability, pyramidal signs, and chronic constipation 35
IAGLI, VDCAL, LLQYG	*NBAS. Neuroblastoma-amplified sequence* Growth retardation, senile face, and optic atrophy 36
GVVFL	*NEUR1. Sialidase-1* Sialidosis: cherry red macular spots in childhood, progressive debilitating myoclonus, insidious visual loss 37
VCGPK, NASVV	*NPC1. NPC intracellular cholesterol transporter 1* Infantile Niemann–Pick type C disease 38
**LVLLPL**	*NPT2A. Sodium-dependent phosphate transport protein 2A* Hypercalcemia, failure to thrive, vomiting, nephrocalcinosis 39
GGFNF, AGAAA	*NUP62. Nuclear pore glycoprotein p62* Infantile bilateral striatal necrosis 40
EMIAQ, LVDLP	*OPA1. Dynamin-like 120 kDa protein, mitochondrial* Lethal encephalopathy, cardiomyopathy optic atrophy 41
KSFTV	*PCD19. Protocadherin-19* *S* eizure, cognitive impairment, and delayed development of variable severity. Mainly affects females 42
EVRQI, KVTLA	*PEX1. Peroxisome biogenesis factor 1* The peroxisome biogenesis disorders include: Zellweger's syndrome, neonatal adrenoleukodystrophy, infantile Refsum's disease, and rhizomelic chondrodysplasia punctata 43
SASFS, **FLVLLP**	*PEX12. Peroxisome assembly protein 12* See above 43
VLLPL	*PEX26. Peroxisome assembly protein 26* See above 43
**LHSTQD**	*PEX6. Peroxisome assembly factor 2* See above 43
LIAIV	*PIGP. Phosphatidylinositol N-acetylglucosaminyltransferase subunit P* Developmental and epileptic encephalopathy 44
LQPEL	*PRRT2. Proline-rich transmembrane protein 2* Recurrent and brief attacks of abnormal involuntary movements, triggered by sudden voluntary movement 45
QIAPG	*PTH2. Peptidyl-tRNA hydrolase 2, mitochondrial* Global developmental delay, hypotonia, hearing loss, ataxia, hyporeflexia, hypothyroidism, and pancreatic insufficiency 46
DLFLP	*RMND1. Required for meiotic nuclear division protein 1* Neonatal hypotonia and lactic acidosis. Affected individuals may have respiratory insufficiency, seizures 47
KRVDF	*RPB1. DNA-directed RNA polymerase II subunit RPB1* Hypotonia and intellectual and behavioral abnormalities 48
PGDSS	*SC6A3. Sodium-dependent dopamine transporter* Infantile parkinsonism-dystonia 49
NLAAT	*SCN1A. Sodium channel protein type 1 subunit α* Generalized epilepsy with febrile seizures persisting beyond the age of 6 y and/or a variety of afebrile seizure types 50
NLAAT	*SCN2A. Sodium channel protein type 2 subunit α* Benign infantile epilepsy 51
NLAAT	*SCN3A. Sodium channel protein type 3 subunit α* Epilepsy with focal seizures arising from temporal, frontal, parietal, occipital lobes 52
NLAAT, DPLSE	*SCN8A. Sodium channel protein type 8 subunit α* Delayed cognitive and motor development, attention deficit disorder, and cerebellar ataxia 53
VVLSF, NLDSK	*SIAT6. CMP-N-acetylneuraminate-β-1,4-galactoside α-2,3-sialyltransferase* Significantly below average general intellectual functioning associated with impairments in adaptive behavior 54
LQPRT	*SIAT9. Lactosylceramide α-2,3-sialyltransferase* Salt and pepper syndrome with seizures, psychomotor delay, cortical blindness. Patches of skin hypo- or hyperpigmentation 55
QSLLI	*SLF2. SMC5-SMC6 complex localization factor protein 2* Infantile-onset spinocerebellar ataxia 56
GRLQS	*SPTN2. Spectrin β chain, nonerythrocytic 2* Spinocerebellar ataxia 57
**SASFST**	*STXB1. Syntaxin-binding protein 1* Developmental and epileptic encephalopathy 58
FIAGL	*SUCA. Succinate-CoA ligase subunit α, mitochondrial* Infantile onset of hypotonia, lactic acidosis, severe psychomotor retardation, progressive neurologic deterioration 59
LADAG	*SYLC. Leucine–tRNA ligase, cytoplasmic* Infantile liver failure syndrome 60
LPLVS	*SZT2. KICSTOR complex protein SZT2* Developmental and epileptic encephalopathy 61
DSLSS	*TPP1. Tripeptidyl-peptidase 1* Spinocerebellar ataxia 62

Abbreviations: gp, glycoprotein; SARS-CoV-2, severe acute respiratory syndrome coronavirus 2.

a Hexapeptides derived from overlapping pentapeptides are given in bold.

b Human proteins given by UniProt entry and name are in italic. Further details and references on related pathologies are available in PubMed, OMIM, and UniProt databases.

- The unexpectedly high molecular mimicry described in Table 1 and the consequent potential cross-reactivity support the hypothesis that several diseases might occur in children following exposure to the SARS-CoV-2 antigen.
- Mathematically, the vastness of the common molecular platform stands out when one considers that the probability for 2 proteins to share 1 pentapeptide on the basis of the 20 aa and neglecting the relative aa abundance is equal to 1 out of 20 raised to 5. That is, it is equal to 0.0000003125.
- Such unexpected massive peptide commonality between SARS-CoV-2 antigen gp and the human proteome indicates and confirms a strict phenetic relationship between viruses and the origin of eukaryotic cells according to the endosymbiotic theory. 63
- Pathologically, the diseasome that might occur via cross-reactivity includes severe disorders such as nephropathies, seizures, cardiomyopathies, parkinsonism-dystonia, global developmental delay, hypotonia, hearing loss, ataxia, hyporeflexia, hypothyroidism, pancreatic insufficiency, and lethal encephalopathy, inter alia.

### Immunologic Potential of the Peptide Sharing between SARS-CoV-2 Spike gp and Human Proteins Related to Infantile Diseases

The cross-reactivity potential of the peptide sharing described in Table 1 appears to be supported by inspection of IEDB ( www.iedb.org ). 21 Indeed, all the 54 minimal immune determinants common to SARS-CoV-2 spike gp and human proteins related to infantile diseases occur and repeatedly recur in 839 SARS-CoV-2 spike gp–derived epitopes, of which Table 2 displays only a synopsis in the interest of brevity. In essence, Table 2 validates, likely enough, the cross-reactivity hypothesis at the basis of the present study.

**Table 2:** Immunoreactive SARS-CoV-2 spike gp-derived epitopes containing pentapeptides shared between SARS-CoV-2 spike gp and human proteins linked to infantile diseases: a synopsis

IEDB ID a	Epitope sequence b	IEDB ID a	Epitope sequence b
3589	aphGVVFLhv	1325536	tLADAGfik
4321	asaNLAATk	1327418	vydpLQPELdsf
16156	FIAGLIAIV	1327824	wtAGAAAyy
23200	GVVFLhvty	1327836	wtfGAGAAl
36724	litGRLQSl	1329248	dEMIAQytsal
37289	llfnKVTLA	1330227	tqDLFLPff
37724	LLQYGsfct	1330420	aphGVVFL
50166	pyrvVVLSF	1330526	lynSASFSTf
51999	qpyrvVVLSF	1331519	EDLLFn
57592	SEPVLkgvkl	1332003	fvFLVLLPL
57792	sfiEDLLFnk	1332424	itGRLQSlqty
59161	slidLQELGK	1332664	lltdEMIAQy
71996	vydpLQPEL	1332702	LQELGKyeqy
1074967	lepLVDLPi	1332727	ltdEMIAQyt
1075065	stqDLFLPff	1332785	mfvFLVLLPLVSs
1309147	YLQPRTfll	1333450	SASFSTfkcy
1310623	ltdEMIAQy	1333520	SFELLhapatv
1311673	EVRQIAPGqt	1333523	sfiEDLLF
1311846	SFELL	1333568	sKRVDFcgkgy
1313244	nSASFSTfk	1333801	sTECSNLLLQy
1314425	alDPLSEtk	1333812	stqDLFLPf
1315940	epLVDLPi	1333921	tdEMIAQy
1316323	fdeddSEPVL	1334182	vgYLQPRTf
1317916	gYLQPRTfll	1390229	VDCALDPLSEtkctlks
1320443	lgaeNSVAY	1541124	KRVDFcgk
1321078	LPLVSsqcv	1546420	fiEDLLFnk
1322298	NASVVniqk	1547648	gYLQPRTfl
1323200	QELGKyeqy	1593850	YLQPRifll
1323249	QIAPGqtgk	1597683	fiEDLLFnkv
1323750	rasaNLAATk	1625440	ssvLHSTQ
1325401	TECSNLLLQy	1659240	fvFLVLLPLv

Abbreviations: gp, glycoprotein; IEDB, Immune Epitope Database; SARS-CoV-2, severe acute respiratory syndrome coronavirus 2.

a Epitopes listed according to the IEDB ID number. Details and references for each epitope are available at www.iedb.org/. ^21^

b Shared peptides are given capitalized.

### Specificity of the Peptide Sharing between SARS-CoV-2 Spike gp and Human Proteins Linked to Infantile Diseases

To control the specificity of the peptide sharing between SARS-CoV-2 spike gp and human proteins linked to childhood diseases ( Table 1 ), the 54 shared pentapeptides were analyzed for occurrences in other coronaviruses not associated with particular pediatric complications, that is, MERS-CoV, HCoV-OC43, and HCoV-229E. Results are shown in Table 3 that provides evidence that the intense peptide overlap between SARS-CoV-2 spike gp and human proteins related to childhood diseases is highly specific. *De facto* , almost all the 54 shared pentapeptides are absent in the CoV controls, that is, in the pathogenic MERS-CoV 64 as well as in the scarcely pathogenic HCoV-OC43 and HCoV-229E that cause only mild symptoms. 65

**Table 3:** Quantitation of the pentapeptide sharing between CoVs spike gps and human proteins linked to childhood diseases

Spike gp from:	Number of shared pentapeptides
SARS-CoV-2	54
MERS-CoV	−
hCoV-229E	−
hCoV-NL63	2

Abbreviations: CoV, coronavirus; gp, glycoprotein; h, human; MERS, Middle East respiratory syndrome; SARS-CoV-2, severe acute respiratory syndrome coronavirus 2.

### Occurrence in Bacteria of Peptides Common to SARS-CoV-2 Spike gp and Proteins Linked to Infantile Diseases

To further control the specificity of the peptide sharing between SARS-CoV-2 spike gp and human proteins linked to childhood diseases, comparative sequence analyses were extended to the bacterial pathogens *B. pertussis* , *C. diphtheriae* , *C. tetani* , *H. influenzae* , and *N. meningitides* —that is, bacteria to which children may be exposed also following current vaccinal routes—were analyzed. Results are displayed in Table 4 .

**Table 4:** Occurrences in bacterial pathogens of pentapeptides shared between SARS-CoV-2 spike gp and human proteins linked to infantile diseases

Organism	Shared pentapeptides
*B. pertussis*	AGAAA, AGTIT, DIAAR, DSLSS, FIAGL, FLVLL, GAGAA, GVVFL, KVTLA, LADAG, LIAIV, LPLVS, LQELG, LVDLP, NASVV, NLAAT, QELGK, SLLIV, VLLPL
*C. diphtheriae*	AGAAA, AGTIT, GAGAA, LADAG, VPVAI
*C. tetani*	AGAAA, AGTIT, DLFLP, EVRQI, FIAGL, LLQYG, LQELG, NASVV, SSVLN
*H. influenzae*	AGAAA, AGTIT, DIAAR, EVRQI, GAGAA, GGFNF, GVVFL, IAGLI, KRVDF, LADAG, LIAIV, LPLVS, LQELG, LVDLP, LVLLP, NASVV, NLAAT, NLDSK, NLLLQ, NSVAY, QELGK, QIAPG, QSLLI, SFELL, SLLIV, SSVLN, TLLAL, VLLPL, VPVAI, VVLSF
*N. meningitidis*	AGAAA, ASFST, EVRQI, FIAGL, FLVLL, GAGAA, KRVDF, LADAG, LQELG, LQPEL, NLDSK, QELGK, TLLAL, VLLPL, VVLSF

Abbreviations: gp, glycoprotein; SARS-CoV-2, severe acute respiratory syndrome coronavirus 2.

It can be seen that many of the 54 peptides shared between SARS-CoV-2 spike gp and human proteins linked to infantile diseases also occur in the analyzed microbial organisms, thus highlighting that, contrary to expectations, while practically no phenetic similarity exists between SARS-CoV-2 spike gp and the control CoVs ( Table 3 ), a high level of similarity exists between SARS-CoV-2 spike gp and bacterial pathogens to which children have been exposed via passive/active infection and by which the immune system has already been imprinted ( Table 4 ).

Then, as highlighted in the literature, 66 67 68 69 70 71 72 the interpathogen peptide commonality can add a further potential burden to the molecular mimicry phenomenon described in Table 1 . Indeed, a fundamental property of the immune system is the memory for immune determinants previously encountered so that, as a rule, the immune system reacts by recalling memory of the responses toward past infections rather than producing *ex novo* responses toward the recent ones.

Consequently, in the case under study here, the following sequence of events may unfold:

- A primary response to SARS-CoV-2 can actually occur as a secondary (or even tertiary) response against pathogens previously encountered and memorized by the immune system. That is, anamnestic secondary antibacterial responses can occur after exposure to SARS-CoV-2. Such anamnestic secondary antibacterial responses will be of considerable proportions given the extent of the viral versus bacterial peptide overlap described in Table 4 .
- SARS-CoV-2 antigen will not be affected in that the immunologic memory deflects the immune response toward the already encountered peptides, that is, the bacterial peptide platform detailed in Table 4 .
- However, also the attack against the early sensitizing bacterial pathogens can fail by being the early sensitizing bacterial pathogens no more present in the organism.
- Then, the ultimate result might be that the anamnestic, high affinity, high avidity, and extremely potent secondary immune response elicited by the lastly encountered pathogen—that is, SARS-CoV-2—can hit the only available targets, that is, the common immune determinants that in this instance are present in the human proteins related to infantile diseases ( Table 1 ).

According to this sequence of events, molecular mimicry and immunologic memory might explain also the different pathological outcomes of the autoimmune responses—from mild symptoms to even lethal pathologies—that may follow exposure to SARS-CoV-2. In practice, the history of infections/immunization of each child is the main factor dictating the onset and the severity of the pathologies outlined in Table 1 .

## Conclusion

The present study investigates the possible adverse events that might occur in newborns and children following exposure to SARS-CoV-2. Based on the extensive peptide sharing between SARS-CoV-2 gp antigen and human proteins related to infantile diseases, supported by epitopic data that confer a high immunoreactivity to the peptide sharing, and given the possibility of immunologic imprinting phenomena, this study leads to predict that exposing newborns and children to SARS-CoV-2 might associate with infantile severe diseases such as growth retardation, abnormal genitalia, epilepsy, seizures, cardiomyopathies, hypotonia, visual loss, hypercalcemia, ataxia, infantile parkinsonism-dystonia, below average general intellectual functioning, encephalopathies, and inter alia. Then, the present data suggest that extreme caution be exercised in planning and implementing a mass anti-SARS-CoV-2 vaccination of infants and children.
