# Supplementary material for: Exposure to SARS-CoV-2 and Infantile Diseases
Source: Glob Med Genet. 2023 May 2;10(2):72–8. doi: 10.1055/s-0043-1768699 (PMC10154082; doi:10.1055/s-0043-1768699)
Supplement: Supplementary file 1 — Supplementary Material [file 10-1055-s-0043-1768699-s2300011.pdf]

Supplementary Table S1 List of 372 human proteins retrieved from UniProt database using the keyword “infantile”

1433G; A1AT; AATM; ABCB7; ABCBB; ACAD9; ACHA2; ACHE; ACK1; ACL6B; ACON; ACTH; ADA22; AGK; AIFM1; AIMP1; ALAT2; ALG13; ALG14; ALS2; ANKS6; ANKZ1; ANTR1; ANTR2; AP3B2; AP4M1; ARF6; ARHG9; ARSA; ARV1; ARX; ASAH1; ASM; AT1A2; AT1A3; ATP6; ATP8; BCS1; BGAL; BSND; CA2D2; CAC1A; CAC1E; CAC1F; CAH5A; CBPC1; CC174; CCHL; CD59; CDK19; CDKL5; CELR3; CEP83; CH60; CIA30; CLAT; CLCKA; CLCKB; CLCN7; CLN5; CLN6; CLN8; CMC1; CNPY3; CO1A1; CO4A1; COA5; COA6; COQ2; COQ5; COQ7; COQ9; COT1; COX15; CP1B1; CP24A; CPLX1; CPT2; CRYAB; CSCL1; CSKP; CTNS; CUX2; CX6B1; CXA3; CYB; CYFP2; D2HDH; DALD3; DEN5A; DLP1; DMXL2; DNM1L; DNMBP; DOCK7; DPOG1; DPYL2; DSRAD; DYN1; EAA2; ECHP; EF1A2; EFGM; EFL1; EFTU; EI2BA; EI2BB; EI2BD; EI2BE; EI2BG; ENPP1; FAKD2; FBXL4; FGF12; FHL1; FOXN1; FRMD7; FRS1L; FXRD1; GABR2; GALC; GARS; GATA; GBRA1; GBRA2; GBRA5; GBRB1; GBRB2; GBRB3; GBRG2; GCSH; GCSP; GCST; GFAP; GHC1; GLPK; GLSK; GNAO; GP143; GPDA; GRDN; GTR1; GUC2C; GUF1; HAX1; HCD2; HCN1; HECAM; HEXA; HEXB; HIBCH; HIKES; HNRPU; HSF4; HTRA2; IDHP; IL21; INVS; IPYR2; IR3IP; ISCA2; ITPA; KCNA1; KCNA2; KCNB1; KCNQ2; KCNQ3; KCNQ5; KCNT1; KCNT2; KIF3C; KIF5A; KIF5C; LAMA3; LAMB3; LAMC2; LRC33; LYAG; MA2B1; MDHC; MDHM; MECP2; MED17; MEF2C; MFSD8; MIPEP; MPV17; MTM1; MTO1; MTU1; MYL3; MYOC; NACC1; NAGAB; NALCN; NBAS; NDF2; NDP; NECP1; NEK8; NEUR1; NFASC; NLRC4; NLRP3; NMDE2; NMDE4; NMDZ1; NNRE; NOTC3; NPC1; NPC2; NPHP3; NPT2A; NRP2; NTRK2; NU2M; NU3M; NU5M; NU6M; NUP62; OAS1; OPA1; OSTM1; OTUL; OXR1; P53; PACS2; PALM2; PAX7; PCD19; PDE10; PEO1; PEX1; PEX10; PEX12; PEX13; PEX14; PEX16; PEX19; PEX2; PEX26; PEX3; PEX5; PEX6; PEX7; PGFRB; PHAR1; PIGA; PIGB; PIGO; PIGP; PIGQ; PKHD1; PLAP; PLCB1; PLPL9; PNKP; PP2BA; PPBT; PPGB; PPT1; PRRT2; PSPC; PT100; PTH2; PX11B; PYC; PYR1; RENR; RGPA1; RHBT2; RINT1; RM44; RMND1; RNF13; RNT2; RPB1; S12A5; S13A5; S17A5; S19A3; S35A2; S52A2; SAP; SC5A6; SC5AB; SC6A3; SC6A9; SCN1A; SCN1B; SCN2A; SCN3A; SCN8A; SCNBA; SCO1; SCO2; SCYL1; SDHA; SDHF1; SEN54; SERA; SERB; SERC; SIAS; SIAT6; SIAT9; SIK1; SLF2; SMBP2; SMC1A; SMN; SNP25; SOX3; SPAST; SPTN1; SPTN2; SRGP2; STAT3; STB5L; STING; STXB1; SUCA; SUCB1; SYAC; SYAM; SYEM; SYFM; SYIC; SYK; SYLC; SYMC; SYNJ1; SYPM; SYRM; SYT1; SYWM; SZT2; TAZ; TBB2A; TBC24; TBCD; TBCK; TERA; TGFB1; THIOM; TNNT2; TPP1; TRAK1; TREX1; TRIM8; TT21B; TTC37; TY3H; UBA1; UBA5; UGDH; UGPA; UN13C; UNC80; VATA; VGFR2; VGFR3; VMAT2; VPP3; VRK1; WIPI4; WWOX; ZAP70; ZEB2; ZN423; ZNHI3; ZSWM6

Note: Proteins are given by UniProt entry.

 © 2023. The Author(s).
